# Tips for Transitioning to a New Electronic Health Record System

**DOI:** 10.6004/jadpro.2012.3.5.6

**Published:** 2012-09-01

**Authors:** Jean Rosiak

**Affiliations:** From Aurora Health Care, Comprehensive Breast Center, Milwaukee, Wisconsin

The dreaded memo came on a Friday afternoon (as dreaded memos often seem to do). It read: "You must sign up for training for the change from Cerner to the Epic System." Insert any vendor’s name in place of either of these two electronic health record (EHR) systems,1 and the feeling of dread it brings on is the same.

My first question was "Why?" The current system seemed to be working just fine. One of the answers to that question is "financial incentive." The American Recovery and Reinvestment Act of 2009 (ARRA) allocates as much as $27 billion over 10 years to support the adoption of EHRs.

## Financial Incentive

The Centers for Medicare & Medicaid Services (CMS) is responsible for implementing the provisions of ARRA by providing incentives to providers who demonstrate "meaningful use" of certified EHR technology (CMS, 2012). The goal is to improve the quality, safety, and effectiveness of care. The timeline for participation in the incentive program and implementation of an EHR spans 2011 to 2015. Each year, participating providers and hospitals receive an incentive for accomplishing defined goals. Participation must begin by 2012 to receive maximum incentive payment. Beginning in 2015, providers and hospitals that do not demonstrate meaningful use of EHRs will receive a decreased reimbursement rate for treating Medicare patients. Therefore, there is a strong incentive for practices and institutions to participate in the program and comply with the requirements as soon as possible.

Eligible professionals may receive as much as $44,000 over 5 years through Medicare or $63,750 over 6 years through Medicaid. Participation is limited to one program or the other, not both. "Eligible professional" is defined by Medicare as an individual legally authorized to practice under state law as a doctor of medicine, osteopathy, optometry, dental medicine, or surgery, or as a chiropractor (CMS, 2010a). Medicaid also includes nurse practitioners, certified nurse midwives, and physician assistants (CMS, 2010b). Hospitals begin with a base payment of $2 million and after that can increase incentive payments based on a number of factors (e.g., number of discharges).

## What Is Meaningful Use?

Meaningful use implies more than just changing the format of data recording from paper to digital (CMS, 2010c). The CMS has proposed three stages for phasing in changes to demonstrate meaningful use of electronic records:

**Stage 1 (2011 and 2012):** Entering required data in a minimum percentage of records to track information and clinical conditions, and initiating reporting of quality measures and public health information.

**Stage 2 (2013):** Expansion of stage 1 and incorporation of disease management, clinical decision support, e-prescribing, and health information exchange.

**Stage 3 (2015):** Focus on improvements in quality, safety, and efficiency; decision support for national high-priority conditions; improving health outcomes; and patient access to self-management tools.

Providers must meet specified core objectives as well as implement measures from a list of options (Prescriber’s Letter, 2011). Some of the objectives providers and institutions must demonstrate to qualify for incentive payments are as follows:

- Use of computerized provider order entry (CPOE)
- Use of drug-drug and drug-allergy interaction checks
- An accurate and current problem list
- Use of electronic prescribing
- An accurate medication and allergy list
- Record of specified demographics (i.e., preferred language, gender, race, etc.)
- Record of vital signs and smoking status
- Quality measure reporting to the CMS
- Provision of an electronic copy of health records to patients on request
- After-visit summary provided to the patient following each office visit
- Protection of EHR information
- Electronic exchange of important clinical information

The minimum data must be collected and consistent use must be demonstrated to fulfill the "meaningful use" criteria.

## Coping With Change

So how do nurse practitioners and physician assistants who are required to conform to use of "certified EHR technology" survive the process of learning to use the new system? As I have recently been through the transition to a new EHR system, here are some suggestions for those anticipating such a change:

**Take all the training** you are offered, and keep user support numbers handy.

**Allow yourself enough time** to get acquainted with the system: Everything will take longer at first, but you will get faster at it as you become more familiar with the system.

**Play with it:** Try different ways of doing things to see what works best for you.

**Keep an open mind,** and listen to explanations of optional features. A feature you think you won’t use now may turn out to be your favorite aspect of the system once you become a more experienced user.

**Change is not necessarily bad:** Don’t expect it to work like the old system did; some things will be worse, but some will be better. Many of the seemingly redundant or inane tasks are requirements for receiving reimbursement.

**Garbage in, garbage out:** The system depends on everyone doing his or her part, so it is essential to enter information that is accurate and up to date.

**Remember the goal:** Improved communication and safe, efficient, quality care.

**Focus on the patient,** not the computer.

## Conclusion

There is an immense national investment in EHRs, with a goal of developing a nationwide health information network. Decision support and protective safeguards can be included to ensure safe and evidence-based quality care. But in our day-to-day practices, we need to become comfortable with whatever system is being implemented and learn to use it optimally. It will take time and an initial investment, but it will eventually make our work life easier and more efficient. When such systems are used properly, the correct and current information can be available when and where it is needed. As providers, this translates into our ultimate goal: quality care, efficiency, and safety for our patients.
